# Einfluss der beruflichen Tätigkeit auf Erkrankungen des Bewegungsapparates der oberen Extremität

**DOI:** 10.1007/s00132-021-04199-1

**Published:** 2021-12-22

**Authors:** Stefan Hertling, Franziska Loos, Georg Matziolis, Isabella Kirschner, Isabel Graul

**Affiliations:** 1 Klinik und Poliklinik für Frauenheilkunde und Fortpflanzungsmedizin, Universitätsklinikum Jena, Am Klinikum 1, 07747 Jena, Deutschland; 2 Praxis für Orthopädie und Schulterchirurgie, 04177 Leipzig, Deutschland; 3 Waldkliniken Eisenberg, Deutsches Zentrum für Orthopädie, Campus Eisenberg, Universitätsklinikum Jena, Eisenberg, Deutschland; 4 Klinik für Unfall‑, Hand- und Wiederherstellungschirurgie, Universitätsklinikum Jena, Am Klinikum 1, 07747 Jena, Deutschland; 5 Heinrich-Schütz-Straße 16, 07548 Gera, Deutschland

**Keywords:** Beruf, Prävalenz, Retrospektive Studie, Rotatorenmanschette, Schulterschmerzen, Occupation, Prevalence, Retrospective studies, Rotator cuff, Shoulder pain

## Abstract

**Einleitung:**

Erkrankungen des Bewegungsapparates der oberen Extremität sind Grund für zunehmende krankheitsbedingte Fehlzeiten bei Erwerbspersonen.

**Zielsetzung:**

Ziel dieser Studie ist es, den Einfluss der Berufsabhängigkeit auf die Entstehung von Erkrankungen des Bewegungsapparates der oberen Extremität zu untersuchen und neben berufsspezifischen Faktoren, gesundheitsbezogene Risiken darzustellen.

**Material und Methoden:**

Es wurden 1070 Patienten eingeschlossen, bei denen zwischen 2016 und 2019 bei einer Läsion der Rotatorenmanschette (RM) eine operative RM-Rekonstruktion durchgeführt wurde. Die relevanten Daten wurden retrospektiv aus dem Krankenhausinformationssystem dokumentiert. Die Berufszweige der Patienten wurden nach der Klassifikation der Berufe 2010 (KldB 2010) eingeteilt und mit routinemäßig erfassten und anonymisierten, frei verfügbaren Daten (Statistisches Bundesamt, Bundesagentur für Arbeit) verglichen.

**Ergebnisse:**

Von den 1070 Patienten waren 844 Patienten im arbeitsfähigen Alter. Die Altersstruktur der einzelnen Bereiche zeigten keine signifikanten Unterschiede. Anhand der Vergleiche der Patientendaten mit der Bevölkerung ergaben sich signifikant höhere RM-Erkrankungsraten in den Bereichen Land‑, Forst- und Tierwirtschaft sowie Gartenbau (*p* = 0,003); Bau, Architektur, Vermessung und Gebäudetechnik (*p* < 0,001); Verkehr, Logistik, Schutz und Sicherheit (*p* < 0,001) und Unternehmensorganisation, Buchhaltung, Recht und Verwaltung (*p* < 0,001). Ein signifikant reduziertes Risiko bestand in Naturwissenshaft, Geografie und Informatik (*p* = 0,015); kaufmännische Dienstleistungen, Warenhandel, Vertrieb, Hotel und Tourismus (*p* < 0,001); Gesundheit, Soziales, Lehre und Erziehung (*p* < 0,001).

**Schlussfolgerung:**

Die Prävalenz von RM-Läsionen zeigt einen statistischen Zusammenhang zur ausgeführten Berufstätigkeit in Abhängigkeit von den Berufszweigen. Neben der Berufsabhängigkeit spielen geschlechtsspezifische Arbeitsfaktoren eine Rolle. Schulterschmerzen bei Erwerbstätigkeiten sollten differenzierter betrachtet werden. Dadurch sollen gezielt Präventivmaßnahmen eingeleitet werden können, um vorzubeugen.

## Abstract (en)

**Introduction:**

Diseases of the musculoskeletal system of the upper extremity are the reason for increasing sickness-related absenteeism among the working population.

**Objective:**

The aim of this study is to investigate the influence of occupational dependence on the development of musculoskeletal disorders of the upper extremities and to present health-related risks in addition to occupation-specific factors.

**Materials and methods:**

We included 1070 patients who underwent surgical *rotator cuff (RC)* reconstruction for an RC lesion between 2016 and 2019. The relevant data were retrospectively documented from the hospital information system. The patients’ occupations were classified according to the Classification of Occupations 2010 (KldB 2010) and compared with routinely recorded and anonymized freely available data (Federal Statistical Office, Federal Employment Agency).

**Results:**

Of the 1070 patients, 844 were of working age. The age structure of the individual areas showed no significant differences. Based on the comparisons of patient data with the population, significantly higher *RC* *injury* rates were found in agriculture, forestry, animal husbandry and horticulture (*p* = 0,003); construction, architecture, surveying and building services engineering (*p* < 0,001); transport, logistics, protection, and security (*p* < 0.001) and business organization, accounting, law, and administration (*p* < 0,001). There was a significantly reduced risk in science, geography and computer science (*p* = 0.015); commercial services, goods trade, distribution, hotel and tourism (*p* < 0,001); health, social affairs, teaching and education (*p* < 0,001).

**Conclusion:**

The prevalence of RC lesions shows a statistical correlation with the occupation performed depending on the occupational branches. In addition to occupational dependency, gender-specific work factors play a role. Shoulder pain in gainful employment should be considered in a more differentiated way. This should enable preventive measures to be taken in a targeted manner.

## Kurze Hinführung zum Thema

Einen entscheidenden Einfluss auf die Entstehung von Erkrankungen des Bewegungsapparates der oberen Extremität hat der aktuell ausgeübte Beruf. Der Einfluss des Berufs resultiert dabei aus einer Reihe von Faktoren. Naheliegend ist zunächst die Annahme von berufsspezifisch unterschiedlichen gesundheitsbezogenen Risiken als Folge der Belastung am Arbeitsplatz. Bisherige Erkenntnisse in diesem Zusammenhang weisen darauf hin, dass bestimmte Berufszweige einem höheren Erkrankungsrisiko am Arbeitsplatz ausgesetzt sind als andere. Kann die berufliche Belastung so groß sein, dass sie zu einer Krankheit führt, oder spielen dabei andere Aspekte außerhalb des Arbeitslebens eine größere Rolle für die Krankheitsentstehung?

## Einleitung

Das Schultergelenk mit multidirektionaler Beweglichkeit und hoher Bedeutung für die Arm- und Handfunktion spielt eine wichtige Rolle für die Arbeitsfähigkeit [1, 2]. Läsionen der Rotatorenmanschette (RM) sind eine häufige muskuloskelettale Verletzung der oberen Extremität in der arbeitenden Bevölkerung. Hiervon sind 6,60 % der Männer und 8,50 % der Frauen von einer betroffen [3]. Die Folge sind lange Abwesenheitsperioden vom Arbeitsplatz [4, 5]. Rotatorenmanschettenpathologien und Schmerzen der Schulter sind in Finnland die häufigste Ursache für krankheitsbedingte Abwesenheit vom Arbeitsplatz [6, 7]. Hierbei wurde die krankheitsbedingte Abwesenheit anhand des Auftretens und der Dauer der Abwesenheit bei den krankheitsbedingten Fehlzeiten aufgrund von Muskel-Skelett-Erkrankungen definiert. Die krankheitsbedingte Abwesenheit hing in der Arbeit von Pekkala et al. von den geleisteten Arbeitsjahren ab, mit einem Hauptanteil zwischen dem 45. und 65. Lebensjahr. Je höher die Anzahl der geleisteten Arbeitsjahre war, umso länger war die krankheitsbedingte Abwesenheit von Erwerbstätigen aufgrund einer RM-Läsion [6]. Einen entscheidenden Einfluss auf die Entstehung von Erkrankungen des Bewegungsapparates hat der aktuell ausgeübte Beruf. Der Einfluss des Berufs resultiert dabei aus einer Reihe von Faktoren. Naheliegend ist zunächst die Annahme von berufsspezifisch unterschiedlichen gesundheitsbezogenen Risiken als Folge der Belastung am Arbeitsplatz. Zu den arbeitsassoziierten Faktoren gehört u. a. das häufige Tragen von Gewichten, stark wiederholende Arbeiten und Überkopfarbeiten [8]. In der Literatur finden sich wenig Daten, die den Einfluss der beruflichen Tätigkeit auf die Entstehung von RM-Läsionen darstellen [9]. Bisherige Erkenntnisse in diesem Zusammenhang weisen darauf hin, dass bestimmte Berufszweige einem höheren Erkrankungsrisiko am Arbeitsplatz ausgesetzt sind als andere [10–12]. Ziel dieser Studie ist es, den Einfluss der Berufsabhängigkeit auf die Entstehung von RM-Läsionen zu untersuchen und neben berufsspezifischen Faktoren, gesundheitsbezogene Risiken darzustellen.

## Methodik

Die zuständige Ethikkommission der Universität Jena wurde informiert und hatte keine Einwände gegen die retrospektive, monozentrische Auswertung der Studie mit dem Namen PAMO-NUNK-Shoulder-Study (Reg.-Nr.: 2018-1165-Daten). In dieser Studie wurden alle Patienten eingeschlossen, welche eine arthroskopische Rekonstruktion der RM in den Jahren 2016 bis 2019 erhalten haben. Die Patientenauswahl erfolgte anhand der internationalen Klassifikation der Behandlungsmethoden in der Medizin mit ICD-Codes: M75.1 (Läsionen der RM) und S46.0 (Verletzung der Muskeln und der Sehnen der RM). Anhand der dokumentierten Anamnese und Operationsberichte wurden alle Patienten insbesondere im Hinblick auf ihre berufliche Tätigkeit ausgewertet. Es konnten 1070 Patienten eingeschlossen werden. Bei allen Patienten wurde der aktuelle Beruf dokumentiert, sowie Berentung oder Arbeitslosigkeit, Alter und Geschlecht. Weiterhin wurde die gegebenenfalls zugehörige Berufsgenossenschaft (BG) von der BG anerkannten Verletzungen erhoben.

### Die KldB 2010

Die Klassifikation der Berufe 2010, kurz KldB 2010, wurde von der Bundesagentur für Arbeit und dem Institut für Arbeitsmarkt- und Berufsforschung unter Beteiligung des Statistischen Bundesamtes und den betroffenen Bundesministerien sowie Experten der berufskundlichen und empirischen Forschung entwickelt. Diese wurde im Jahr 2011 eingeführt. Die KldB 2010 wurde vollständig neu entwickelt und löst die Klassifizierung der Berufe aus den Jahren 1988 und 1992 ab. Die KldB 2010 bildet die aktuelle Berufslandschaft in Deutschland realitätsnah ab und bietet zugleich eine hohe Kompatibilität zu anderen internationalen Berufsklassifikationen. Dabei werden Berufe in zehn übergeordneten Berufszweigen sektorenorientiert zusammengefasst (Tab. 1). Die Daten der deutschen Bevölkerung von März 2020 sind in Tab. 1 dargestellt.

**Table 1:** 

Berufsbereich	Aktuelle Zahlen 03/2020
*Gesamt:*	33.648.183
Land‑, Forst- und Tierwirtschaft und Gartenbau	500.654
Rohstoffgewinnung, Produktion und Fertigung	7.218.186
Bau, Architektur, Vermessung und Gebäudetechnik	2.019.667
Naturwissenschaft, Geografie und Informatik	1.357.948
Verkehr, Logistik, Schutz und Sicherheit	4.499.345
Kaufmännische Dienstleistungen, Warenhandel, Vertrieb, Hotel und Tourismus	3.971.079
Unternehmensorganisation, Buchhaltung, Recht und Verwaltung	6.819.708
Gesundheit, Soziales, Lehre und Erziehung	6.181.809
Sprach‑, Literatur‑, Geistes‑, Gesellschafts- und Wirtschaftswissenschaften, Medien, Kunst, Kultur und Gestaltung	892.605
Keine Angabe	187.182

### Daten für Deutschland

Nach Aussage des statistischen Bundesamtes belief sich die Einwohnerzahl in Deutschland im März 2020 auf 83 Mio. Einwohner, wovon 54 Mio. im erwerbsfähigen Alter (15–65 Jahre) waren, 33 Mio. gingen einer sozialversicherungspflichtigen Tätigkeit nach [13]. Bei der Betrachtung der Wirtschaftssektoren befanden sich 2017 lediglich 1,4 % der Erwerbstätigen im primären Sektor (Land- und Forstwirtschaft, Fischerei), wohingegen im sekundären Sektor (produzierendes Gewerbe) 24,1 % eine Beschäftigung fanden. Mit 74,5 % war 2017 der Dienstleistungssektor am stärksten vertreten. Die Gewichtung dieser Bereiche lässt sich durch den strukturellen Wandel der Gesellschaft, welcher sich durch beispielsweise veränderte Nachfrage und zunehmende Automatisierung erklärt, verstehen. Im Dienstleistungssektor ist der Bereich öffentliche Dienstleister, Erziehung, Gesundheit mit 10,9 Mio. Erwerbstätigen (24,7 %) am stärksten gewichtet. Ähnlich viele Personen (10,1 Mio./22,8 %) finden im Wirtschaftsbereich Handel, Verkehr und Gastgewerbe eine Beschäftigung. Nicht zu vernachlässigen ist das Gebiet der Finanzierung, Immobilien, Unternehmensdienstleister mit immerhin 17,4 % aller Erwerbstätigen [14]. Die Bundesagentur für Arbeit gruppierte die Erwerbstätigen in Deutschland anhand der Klassifikation der Berufe 2010. Die Daten der deutschen Bevölkerung von März 2020 sind in Tab. 1 dargestellt [15, 16].

### Studienvergleichsparameter

Anhand der KldB 2010 wurden die dokumentierten Berufe der Patienten eingeteilt. Hierdurch wird eine einheitliche Berufsklassifikation innerhalb dieser Arbeit geschaffen, die statistische Vergleiche zur Gesamtbevölkerung ermöglichen. Diese Daten dienen als Vergleichsgrundlage der Studienergebnisse mit denen von der KldB 2010 aus dem Jahr 2020.

### Datenerhebung

Die Datenerhebung wurde mittels Microsoft Excel 365 (Fa. Microsoft, Redmond, WA, USA) und die statistische Analyse mittels des Statistikprogramms SPSS (Version 22.0, SPSS Inc., Chicago, IL, USA) durchgeführt. Die in der Studie dokumentierten Datensätze wurden mit routinemäßig erfassten und anonymisierten, frei verfügbaren Daten des Statistischen Bundesamtes verglichen. Diese Datensätze sind digital und frei verfügbar auf der Internetseite zugänglich unter der Kategorie: Themen/Arbeit/Arbeitsmarkt/Erwerbstätigkeit [17]. Die deskriptiven Statistiken umfassen Mengen, Prozentsätze, Medianwerte und Bereiche für ordinäre Variablen. Die Verteilung der Daten wurden auf die Normalverteilung untersucht.

Der Chi-Quadrattest wurde für die Analyse von Einflussparametern genutzt und zum Vergleich der Studiendaten mit den Daten des statistischen Bundesamtes eingesetzt. Der Kruskal-Wallis-Test wurde spezifisch zur Erhebung der Altersverteilung in den Berufsgruppen verwendet. Der *p*-Wert von weniger als 0,05 wurde als statistisch signifikant angesehen*.*

## Ergebnisse

### Epidemiologische Daten

**Figure 1:**
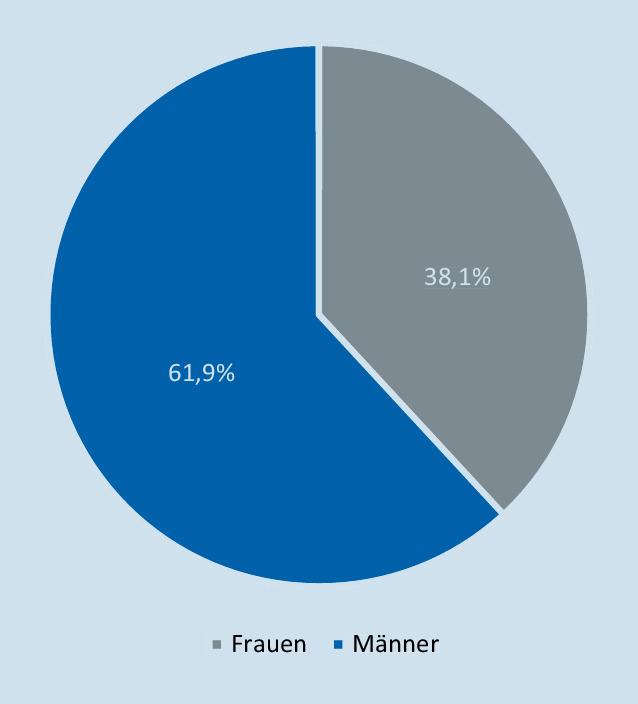

Die Probanden setzten sich aus 662 (61,9 %) Männer und 408 (38,1 %) Frauen zusammen (Abb. 1).

### Altersverteilung

**Figure 2:**
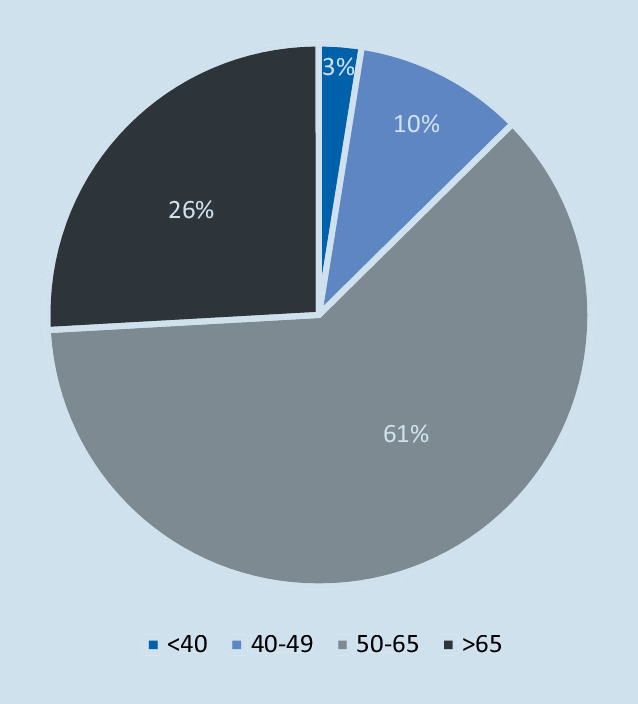

Im Patientenkollektiv waren 26 % älter als 65 Jahre. Im Alter zwischen 50 und 65 waren 61 % und 10 % waren zwischen 40 und 49 Jahre alt. Lediglich 3 % der Patienten, welche eine RM-Rekonstruktion erhielten, waren jünger als 40 Jahre (Abb. 2).

Das Durchschnittsalter lag bei 59,52 Jahren (18–97 Jahre, Median 60 Jahre). Der Altersdurchschnitt betrug für die Männer 59 (±8,33) Jahre und für die Frauen 61 (±8,15) Jahre.

### Vergleich Altersverteilung Studienpopulation und KldB 2010 (2020)

**Figure 3:**
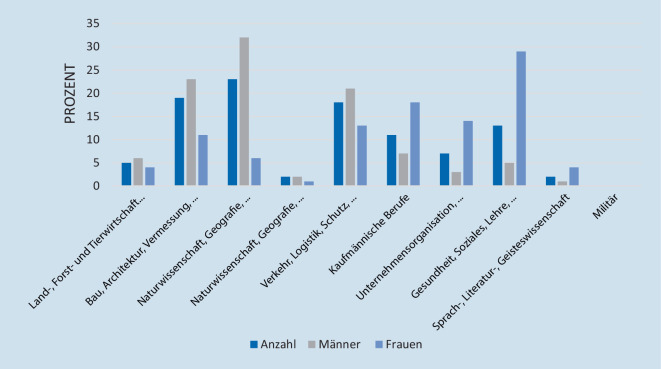

Bei der Altersstruktur zwischen den einzelnen Bereichen unserer Studienpopulation nach den aktuellen Daten der KldB 2010 (2020) ließ sich kein signifikanter Unterschied nachweisen (Abb. 3).

### Erwerbsstatus und Geschlecht

Es waren 844 Patienten im arbeitsfähigen Alter (< 65 Jahre), hiervon gingen 597 einer Beschäftigung nach. Aufgrund von Elternzeit, Arbeitslosigkeit oder EU-Rente gingen 247 keiner Tätigkeit nach. Unter den Berufstätigen fanden sich rund 65 % Männer und 35 % Frauen. Der Altersdurchschnitt der aktuell Beschäftigten lag bei Männern bei 54 Jahren und 55 Jahren bei Frauen. Acht Befragte machten widersprüchliche Angaben, welchen Beruf sie ausübten und konnten nicht eingeschlossen werden (Tab. 2).

**Table 2:** 

	Anzahl	Alter
Land‑, Forst- und Tierwirtschaft und Gartenbau	31	55
Rohstoffgewinnung, Produktion und Fertigung	112	53
Bau, Architektur, Vermessung und Gebäudetechnik	133	55
Naturwissenschaft, Geografie und Informatik	10	56
Verkehr, Logistik, Schutz und Sicherheit	105	55
Kaufmännische Dienstleistungen, Warenhandel, Vertrieb, Hotel und Tourismus	66	55
Unternehmensorganisation, Buchhaltung, Recht und Verwaltung	42	54
Gesundheit, Soziales, Lehre und Erziehung	79	55
Sprach‑, Literatur‑, Geistes‑, Gesellschafts- und Wirtschaftswissenschaften, Medien, Kunst, Kultur und Gestaltung	11	58
Militär	0	0

### Erwerbsstatus, Geschlecht, RM-Läsion im Vergleich zur erwerbstätigen Gesamtbevölkerung nach KldB 2010 (2020)

**Figure 4:**
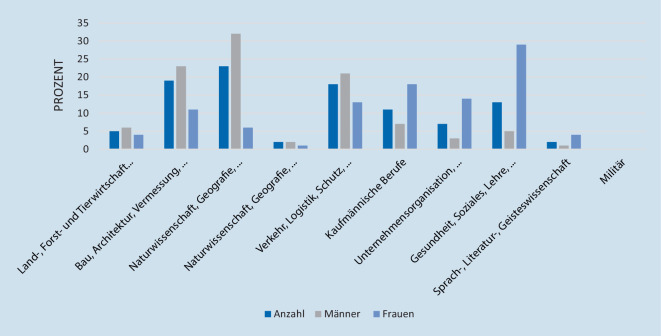

In der Patientenklientel zeigte sich eine signifikante Häufung von Männern (m = 382, w = 207, *p* < 0,001). Nach der Klassifikation der Berufe entfielen auf den Bereich Bau, Architektur, Vermessung und Gebäudetechnik die meisten Arbeitnehmer mit RM-Läsionen mit 23 % (133/589) und den meisten männlichen Arbeitenden mit 32 % (120/382), gefolgt von Rohstoffgewinnung, Produktion und Fertigung mit 19 % (*n* = 112/589) und dem zweithöchsten Männeranteil mit 23 % (89/382), auf Platz 3 folgte der Bereich Verkehr, Logistik, Schutz und Sicherheit mit 18 % (105/589) und dem dritthöchsten Männeranteil mit 21 % (80/382). Der höchste Frauenanteil mit RM-Läsion fand sich im Bereich Gesundheit, Soziales, Lehre und Erziehung. Die Altersstruktur der Gruppen unterteilt nach Klassifikation der Berufe zeigte keine signifikanten Unterschiede (*p* = 0,493). Eine Auflistung wird in Tab. 2 und Abb. 4 dargestellt.

### RM-Läsionen in der Studienpopulation im Vergleich zur Gesamtbevölkerung nach KldB 2010 (2020)

Verglichen mit der Gesamtbevölkerung (Tab. 1) zeigen sich Unterschiede in der Häufigkeit von RM-Läsionen in Bezug auf die Häufigkeit der entsprechenden Berufsklassifikationen (Tab. 3). 25 % der Erwerbstätigen mit sozialversicherungspflichtigem Beruf gingen einer Tätigkeit aus dem Bereich kaufmännische Dienstleistungen, Warenhandel, Vertrieb, Hotel und Tourismus nach, hingegen zeigten in unserem Patientenklientel nur 11 % eine RM-Läsion. In den Berufen Bau, Architektur, Vermessung und Gebäudetechnik arbeiten 9 % der Bevölkerung, allerdings entfielen 23 % der RM-Läsionen unseres Patientenklientels auf diese Berufszweige. Anhand der Vergleiche der Patienten mit der Bevölkerung ergab sich eine signifikant höhere Erkrankungsrate in den Bereichen Land‑, Forst- und Tierwirtschaft und Gartenbau, Bau, Architektur, Vermessung und Gebäudetechnik, Verkehr, Logistik, Schutz und Sicherheit und Unternehmensorganisation, Buchhaltung, Recht und Verwaltung. Ein signifikant reduziertes Risiko bestand in den Berufszweigen Naturwissenshaft, Geografie und Informatik, kaufmännische Dienstleistungen, Warenhandel, Vertrieb, Hotel und Tourismus, Gesundheit, Soziales, Lehre und Erziehung und Sprach‑, Literatur‑, Geistes‑, Gesellschafts- und Wirtschaftswissenschaften, Medien, Kunst, Kultur und Gestaltung.

**Table 3:** 

	Gesamte Bevölkerung in %	Mit Rotatorenmanschettenläsion in %	*p*-Wert
Land‑, Forst- und Tierwirtschaft und Gartenbau	3	5	*p* = 0,003*
Rohstoffgewinnung, Produktion und Fertigung	21	19	*p* = 0,312
Bau, Architektur, Vermessung und Gebäudetechnik	9	23	*p* < 0,001*
Naturwissenschaft, Geografie und Informatik	4	2	*p* = 0,015*
Verkehr, Logistik, Schutz und Sicherheit	10	18	*p* < 0,001*
Kaufmännische Dienstleistungen, Warenhandel, Vertrieb, Hotel und Tourismus	25	11	*p* < 0,001*
Unternehmensorganisation, Buchhaltung, Recht und Verwaltung	5	7	*p* < 0,001*
Gesundheit, Soziales, Lehre und Erziehung	19	13	*p* < 0,001*
Sprach‑, Literatur‑, Geistes‑, Gesellschafts- und Wirtschaftswissenschaften, Medien, Kunst, Kultur und Gestaltung	3	2	*p* = 0,091*
Militär	1	0	*p* = 0,212

*Signifikanter Unterschied

### Einfluss von Geschlecht in Abhängigkeit von KldB 2010 (2020)

Bei der Bildung von Untergruppen nach dem Geschlecht zeigten sich für Männer in den Berufszweigen Bau, Architektur, Vermessung und Gebäudetechnik sowie Verkehr, Logistik, Schutz und Sicherheit signifikant höhere Erkrankungsraten*.* Im Gegensatz dazu arbeiteten Frauen häufiger in den Berufsgruppen kaufmännische Dienstleistungen, Warenhandel, Vertrieb, Hotel und Tourismus sowie im Bereich Gesundheit, Soziales, Lehre und Erziehung, welche ein signifikant geringeres Risiko für RM-Läsionen aufweisen.

### RM-Läsionen im GUV-Verfahren

Von den 589 Patienten mit RM-Läsionen wurden 10 % (61 Fälle) von der zugehörigen Berufsgenossenschaft anerkannt, 84 % der anerkannten Fälle waren männliche Arbeitende. Betroffen waren, abgestuft nach Häufigkeit, die Berufsgruppen Handel und Verkehr (31 %; 19/61), Baugewerbe (25 %; 15/61) und sonstige Dienstleistungen (20 %; 12/61).

## Diskussion

Die Berufsabhängigkeit kann auf die Entstehung von Erkrankungen des Bewegungsapparates der oberen Extremität einen Einfluss haben. Neben berufsspezifischen Faktoren lassen sich gesundheitsbezogene Risiken darstellen. Das Geschlecht eines Erwerbtätigen kann innerhalb einer Berufsgruppe Auswirkungen für das Ausmaß von Erkrankungen des Bewegungsapparates der oberen Extremität und die Reaktionen des Körpers auf bestimmte Arbeitsabläufe und somit das Erkrankungsrisiko haben. Es existieren einige Studien mit der Frage, wann nach einer RM-Läsion oder deren operativen Versorgung eine Rückkehr an den Arbeitsplatz möglich ist [18, 19]. Analysen unter Berücksichtigung anerkannter Berufsklassifikationssysteme zu arbeitsassoziierten Faktoren, welche zu einer RM-Läsion führen oder epidemiologische Untersuchungen zum Auftreten von RM-Läsionen und dem ausgeübten Beruf sind in der Literatur wenig beschrieben.

In der Literatur zum Thema arbeitsassoziierte Faktoren und muskuloskelettale Erkrankungen der oberen Extremität sind meist verschiedene Erkrankungen zusammengefasst, bei denen die RM-Läsion einen hohen Stellenwert hat [20, 21]. In dem Vergleich der Häufigkeiten von Berufsgruppen innerhalb der erwerbsfähigen Bevölkerung und der Häufigkeit der Berufe in der Patientengruppe mit RM-Läsion im erwerbsfähigen Alter zeigt sich eine erhöhte Prävalenz für bestimmte berufliche Belastungen. Aus der Literatur war der Zusammenhang von RM-Läsionen zu Zwangshaltungen mit erhobenen Armen und Überkopfarbeiten [22], für repetitive monotone Arbeiten in Flexion und Abduktion mit Werkzeugen [23], für das Anheben mittlerer und schwerer Lasten über Schulterniveau, für das Ziehen oder Schieben von Gewichten mit den Armen und Vibrationen [24] bekannt. Somit ist bei Berufszweigen mit häufigem Ausführen schulterbelastender Tätigkeiten eine höhere Erkrankungsrate von RM-Läsionen zu erwarten. Dies korreliert stark mit den Ergebnissen, dass die häufigsten RM-Läsionen im Vergleich zur Häufigkeit des Berufszweiges in den körperlich schwereren Tätigkeitsbereichen Bau, Architektur, Vermessung und Gebäudetechnik sowie Verkehr, Logistik, Schutz und Sicherheit zu verzeichnen waren. In der KldB 2010 (2020) waren die Berufszweige erfasst und liefern Anhaltspunkte, dass RM-Verletzungen auch über den allgemeinen degenerativen Prozess durch die Berufstätigkeit beeinflusst werden können. Die hier durchgeführte retrospektive Studie deckt signifikante Zusammenhänge zu bestimmten Berufszweigen auf.

Der Faktor Alter spielte in der vorliegenden Studie als Einflussparameter keine Rolle, da es keine signifikanten Unterschiede im Alter in den Berufszweigen gab. Dies zeigte sich in vorangegangenen internationalen Studien divergent [25–27].

Das Geschlecht als gesundheitsbezogenes Risiko im Kontext zu RM-Läsionen in diesem Zusammenhang spielte bisher in wissenschaftlichen Fragestellungen eine untergeordnete Rolle. Rolf et al. untersuchten mit ihrer Studie, ob Läsionen der RM eine Berufserkrankung seien [6]. Die Daten dieser Studie deuteten darauf hin, dass die berufsbedingte Exposition das Risiko einer RM-Läsion erhöht oder zu einer klinischen RM-Ruptur führen kann. Alleinig wurden hierfür 472 Männer eingeschlossen, sodass im weiterführenden Kontext geschlechtsspezifische Faktoren im berufsspezifischen Kontext in dieser Studie keine Rolle spielten. Weitere internationale Studien zu diesem Thema zeigen ähnliche Inhalte [28, 29]. In einer Studie von van Dyck et al. wurden geschlechtsspezifische Faktoren im beruflichen Kontext bei der Entstehung von RM-Läsionen beschrieben [31]. Van Dyck et al. zeigten auf, dass es im Gesundheitssektor bei den berufsfeldspezifischen Krankenständen aufgrund einer RM-Läsion unter Frauen ein höheres Niveau gab als bei den männlichen Kollegen. Aus diesen Daten resultiert die Annahme, dass neben der Berufsabhängigkeit auch weitere gesundheitsspezifische Risiken bei der Entstehung von RM-Läsionen einen Einfluss haben können [32].

Naheliegend ist die Annahme von berufsspezifisch unterschiedlichen gesundheitsbezogenen Risiken als Folge der Belastung am Arbeitsplatz. So sind Erwerbstätige bestimmter Berufssektoren einem höheren Verletzungsrisiko am Arbeitsplatz ausgesetzt als andere [33]. Der Beruf spielt aber auch insofern eine Rolle, als dass die Tätigkeitsausübung bei ein und derselben gesundheitlichen Einschränkung berufsabhängig unterschiedlich stark beeinträchtigen und somit spezifische sekundäre gesundheitsbezogene Risiken nach sich ziehen kann [26]. So zeigen sich bei Personen mit einer RM-Läsion, die in einem Berufssektor mit wenig Belastung der oberen Extremität tätig sind, eine geringere Gesamtdauer der Arbeitsunfähigkeit im Vergleich zu Personen, deren berufliche Ausübung mit langen oder starken Belastungen der oberen Extremität verbunden ist. Hierbei liegt die Differenz der Dauer der Arbeitsunfähigkeit bei mehreren Wochen [10]. 

Weitere, zum Teil in unterschiedliche Richtungen und nicht ausschließlich berufsgruppenspezifisch wirkende Einflüsse entstehen durch Selektionseffekte oder durch nur mittelbar gesundheitsrelevante Berufsbedingungen. Dazu gehört der sogenannte „healthy worker effect“. Bei Anstellung von körperlich überdurchschnittlich gesunden Personen für besonders belastende Tätigkeiten, können trotz hoher Belastung in bestimmten Berufsgruppen geringe Erkrankungsraten resultieren. Zudem können durch Möglichkeiten zur vorzeitigen Berentung und Einflüsse von tariflich unterschiedlich vereinbarten Entgeltfortzahlungen im Krankheitsfall Selektionseffekte entstehen [34]. Aber auch arbeitnehmerabhängige interindividuelle Faktoren, wie persönliche Kompetenz und Verantwortlichkeit im ausgeübten Beruf, können einen Einfluss auf die Entstehung von Erkrankungen der oberen Extremität haben. Berufs- und zeitabhängig unterschiedlich wahrgenommene Gefahren des Arbeitsplatzverlusts sowie die Berufszufriedenheit und das Arbeitsklima können weiter auf die Entstehung von RM-Läsionen einen Einfluss haben [35]. Die in der Literatur beschriebenen Zusammenhänge aus belastenden Tätigkeiten der oberen Extremität und die epidemiologische Verteilung der vorliegenden Studie zu RM-Läsionen in den Berufszweigen führt zu der Vermutung einer Häufung der schulterbelastenden Tätigkeiten insbesondere im Berufszweig Bau, Architektur, Vermessung und Gebäudetechnik sowie Verkehr, Logistik, Schutz und Sicherheit. Diese Bereiche sollten auf die schulterbelastenden Tätigkeiten genauer untersucht werden, um prädisponierende Faktoren zu definieren und gegebenenfalls auf Prävention oder auch die Definition einer Berufserkrankung abzielen zu können.

## Limitation

Limitierend waren die fehlenden Informationen, wie lange die berufliche Tätigkeit ausgeführt wurde, und die Historie zur beruflichen Tätigkeit der Patienten. Weiterhin fehlten die Informationen zu den einzelnen belastenden Tätigkeiten pro Arbeitstag, wie zum Beispiel Überkopfarbeiten, repetitive Arbeiten oder die Belastung durch Vibrationen. Ein Selektionsbias dieser Studie liegt insofern vor, dass Patienten mit nicht mehr rekonstruierbarer oder konservativ behandelter RM-Läsion nicht in die Analyse einbezogen werden konnten. In der Studie erfolgte keine weitere Unterteilung der RM-Läsionen nach Grad der Verfettung oder nach beteiligten Sehnen und Rissarten. Weiterhin wurde die Histologie nicht erfasst, um Rückschlüsse auf die Pathologie der Läsion zu ziehen. Eine vollständige Diskussion der berufsgruppenspezifischen Berufsabhängigkeit auf die Entstehung von Erkrankungen des Bewegungsapparates der oberen Extremität muss all diese Einflussmöglichkeiten abwägen. Allerdings zeigen sich bei einer Betrachtung von entsprechenden Auswertungsergebnissen Muster, die sich auch ohne den Anspruch einer vollständigen Diskussion sinnvoll interpretieren lassen können. Diese wissenschaftliche Arbeit behandelt eine interessante sozioökonomische Fragestellung in Bezug auf die Entstehung von RM-Läsionen.

## Fazit für die Praxis

- Der Beruf kann einen Einfluss auf die Entstehung von Rotatorenmanschetten(RM)-Läsionen haben.
- Spezifische sekundäre gesundheitsbezogene Risiken können ebenfalls einen Einfluss auf die Entstehung von RM-Läsionen haben.
- Zusammenhänge aus den belastenden Tätigkeiten der oberen Extremität und die epidemiologische Verteilung der vorliegenden Studie zu RM-Läsionen in den Berufszweigen führen zu der Vermutung einer Häufung der schulterbelastenden Tätigkeiten.
- Berufssektoren mit schulterbelastenden Tätigkeiten sollten genauer untersucht werden, um prädisponierende Faktoren zu definieren und gegebenenfalls auf die Prävention oder auch die Definition einer Berufserkrankung abzielen zu können.
- Symptome der oberen Extremität bei Betroffenen, die in prädestinierten Berufszweigen arbeiten, sollten differenziert betrachtet werden.

## Abkürzungen

BG: Berufsgenossenschaft
GUV: Gesetzliche Unfallversicherung
ICD: International Statistical Classification of Diseases and Related Health Problems
KldB: Klassifikation der Berufe
RM: Rotatorenmanschette
